# Methyl 5-chloro-2-(4-methyl­benzene­sulfonamido)­benzoate

**DOI:** 10.1107/S1600536810019045

**Published:** 2010-05-26

**Authors:** Bin Wang, Song Xia, Ya-Bin Shi, Fei-Fei He, Hai-Bo Wang

**Affiliations:** a College of Light Industry and Food Science, Nanjing University of Technology, Xinmofan Road No. 5 Nanjing, Nanjing 210009, People’s Republic of China; b College of Science, Nanjing University of Technology, Xinmofan Road No. 5 Nanjing, Nanjing 210009, People’s Republic of China

## Abstract

In the title compound, C_15_H_14_ClNO_4_S, the benzene rings are oriented at a dihedral angle of 85.42 (1)°. An intra­molecular N—H⋯O hydrogen bond results in the formation of a five-membered ring and an intramolecular C—H⋯O inter­action also occurs.

## Related literature

For general background to the use of the title compound as an inter­mediate in the synthesis of quinoline, see: Theeraladanon *et al.* (2004 ▶). For bond-length data, see: Allen *et al.* (1987 ▶).
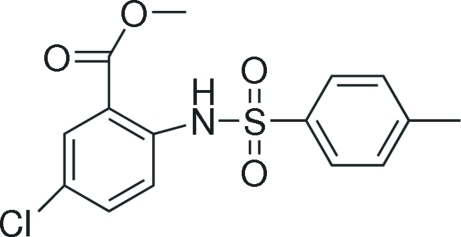


## Experimental

### 

#### Crystal data

C_15_H_14_ClNO_4_S
*M* *_r_* = 339.78
Monoclinic, 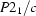

*a* = 18.549 (4) Å
*b* = 9.935 (2) Å
*c* = 8.5190 (17) Å
β = 97.34 (3)°
*V* = 1557.1 (5) Å^3^
*Z* = 4
Mo *K*α radiation
μ = 0.40 mm^−1^
*T* = 293 K
0.30 × 0.10 × 0.05 mm

#### Data collection

Enraf–Nonius CAD-4 diffractometer
Absorption correction: ψ scan (North *et al.*, 1968 ▶) *T*_min_ = 0.891, *T*_max_ = 0.981
2914 measured reflections
2825 independent reflections
1259 reflections with *I* > 2σ(*I*)
*R*_int_ = 0.093
3 standard reflections every 200 reflections  intensity decay: 1%

#### Refinement

*R*[*F* ^2^ > 2σ(*F* ^2^)] = 0.067
*wR*(*F* ^2^) = 0.141
*S* = 1.00
2825 reflections
200 parameters
H-atom parameters constrained
Δρ_max_ = 0.45 e Å^−3^
Δρ_min_ = −0.29 e Å^−3^

### 

Data collection: *CAD-4 EXPRESS* (Enraf–Nonius, 1989 ▶); cell refinement: *CAD-4 EXPRESS*; data reduction: *XCAD4* (Harms & Wocadlo, 1995 ▶); program(s) used to solve structure: *SHELXS97* (Sheldrick, 2008 ▶); program(s) used to refine structure: *SHELXL97* (Sheldrick, 2008 ▶); molecular graphics: *SHELXTL*; software used to prepare material for publication: *PLATON* (Spek, 2009 ▶).

## Supplementary Material

Crystal structure: contains datablocks global, I. DOI: 10.1107/S1600536810019045/bq2211sup1.cif

Structure factors: contains datablocks I. DOI: 10.1107/S1600536810019045/bq2211Isup2.hkl

Additional supplementary materials:  crystallographic information; 3D view; checkCIF report

## Figures and Tables

**Table 1:** Hydrogen-bond geometry (Å, °)

*D*—H⋯*A*	*D*—H	H⋯*A*	*D*⋯*A*	*D*—H⋯*A*
N—H1⋯O4	0.86	2.07	2.615 (6)	120
C9—H9*A*⋯O2	0.93	2.35	3.022 (6)	129

**Fig. 1.**
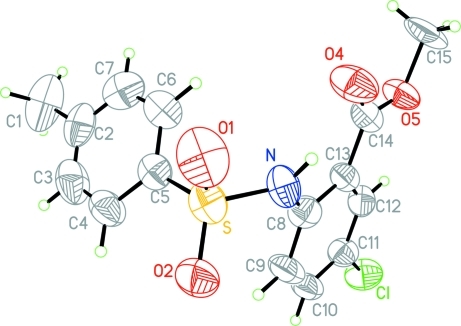
The molecular structure of the title molecule, with the atom-numbering scheme. Displacement ellipsoids are drawn at the 50% probability level.

**Fig. 2.**
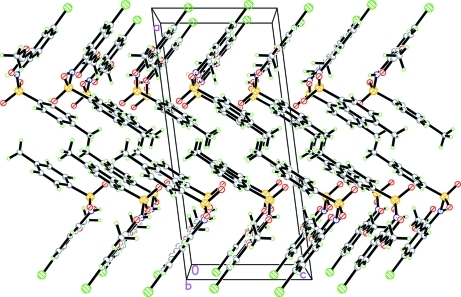
A packing diagram of (I).

## supplementary crystallographic information

### Comment

Quinolines are a major class of alkaloids and play an important role in the fields of natural products and medicinal chemistry. The title compound, (I), is a useful intermediate. (Theeraladanon *et al.*, 2004) and we report here in the crystal structure of it. In the molecule of the title compound (Fig. 1), the bond lengths (Allen *et al.*, 1987) and angles are within normal ranges. Rings A(C2-C7) and B(C8-C13) are planar with a dihedral angle of 85.42 (1) ° between them. The intramolecular N-H···O hydrogen bond (Table 1) results in the formation of a five-membered ring C (C8/C13/C14/O4/N). In the crystal structure, intermolecular C-H···O hydrogen bonds link the molecules into chains along the *b* axis (Fig. 2).

### Experimental

The title compound, (I) was prepared by the literature method (Theeraladanon *et al.*, 2004). Crystals suitable for X-ray analysis were obtained by slow evaporation of an methanol solution.

### Refinement

H atoms were positioned geometrically, with N-H = 0.86 Å (for NH) and C-H = 0.93, 0.98 and 0.96 Å for aromatic, methine and methyl H, respectively, and constrained to ride on their parent atoms, with U_iso_(H) = xU_eq_(C,N), where x = 1.5 for methyl H and x = 1.2 for all other H atoms.

### Crystal data

**Table:**

C_15_H_14_ClNO_4_S	*F*(000) = 704
*M**_r_* = 339.78	*D*_x_ = 1.449 Mg m^−^^3^
Monoclinic, *P*2_1_/*c*	Melting point: 388 K
Hall symbol: -P 2ybc	Mo *K*α radiation, λ = 0.71073 Å
*a* = 18.549 (4) Å	Cell parameters from 25 reflections
*b* = 9.935 (2) Å	θ = 8–12°
*c* = 8.5190 (17) Å	µ = 0.40 mm^−^^1^
β = 97.34 (3)°	*T* = 293 K
*V* = 1557.1 (5) Å^3^	Needle, colourless
*Z* = 4	0.30 × 0.10 × 0.05 mm

### Data collection

**Table:**

Enraf–Nonius CAD-4 diffractometer	1259 reflections with *I* > 2σ(*I*)
Radiation source: fine-focus sealed tube	*R*_int_ = 0.093
graphite	θ_max_ = 25.3°, θ_min_ = 1.1°
ω/2θ scans	*h* = 0→22
Absorption correction: ψ scan (North *et al.*, 1968)	*k* = 0→11
*T*_min_ = 0.891, *T*_max_ = 0.981	*l* = −10→10
2914 measured reflections	3 standard reflections every 200 reflections
2825 independent reflections	intensity decay: 1%

### Refinement

**Table:**

Refinement on *F*^2^	Primary atom site location: structure-invariant direct methods
Least-squares matrix: full	Secondary atom site location: difference Fourier map
*R*[*F*^2^ > 2σ(*F*^2^)] = 0.067	Hydrogen site location: inferred from neighbouring sites
*wR*(*F*^2^) = 0.141	H-atom parameters constrained
*S* = 1.00	*w* = 1/[σ^2^(*F*_o_^2^) + (0.033*P*)^2^] where *P* = (*F*_o_^2^ + 2*F*_c_^2^)/3
2825 reflections	(Δ/σ)_max_ < 0.001
200 parameters	Δρ_max_ = 0.45 e Å^−^^3^
0 restraints	Δρ_min_ = −0.29 e Å^−^^3^

### Special details

**Table:**

Geometry. All esds (except the esd in the dihedral angle between two l.s. planes) are estimated using the full covariance matrix. The cell esds are taken into account individually in the estimation of esds in distances, angles and torsion angles; correlations between esds in cell parameters are only used when they are defined by crystal symmetry. An approximate (isotropic) treatment of cell esds is used for estimating esds involving l.s. planes.
Refinement. Refinement of F^2^ against ALL reflections. The weighted R-factor wR and goodness of fit S are based on F^2^, conventional R-factors R are based on F, with F set to zero for negative F^2^. The threshold expression of F^2^ > 2sigma(F^2^) is used only for calculating R-factors(gt) etc. and is not relevant to the choice of reflections for refinement. R-factors based on F^2^ are statistically about twice as large as those based on F, and R- factors based on ALL data will be even larger.

### Fractional atomic coordinates and isotropic or equivalent isotropic displacement parameters (Å^2^)

**Table:**

	*x*	*y*	*z*	*U*_iso_*/*U*_eq_	
S	0.29259 (8)	0.49679 (16)	1.24335 (16)	0.0564 (4)	
Cl	−0.02197 (9)	0.34294 (17)	0.71715 (19)	0.0780 (6)	
O1	0.3413 (2)	0.4520 (4)	1.3779 (4)	0.0819 (14)	
O2	0.2559 (2)	0.6236 (4)	1.2478 (5)	0.0722 (12)	
O4	0.2340 (2)	0.1125 (4)	1.2061 (5)	0.0770 (13)	
O5	0.1500 (2)	0.0066 (4)	1.0409 (4)	0.0642 (11)	
N	0.2329 (2)	0.3752 (4)	1.2181 (5)	0.0583 (13)	
H1	0.2374	0.3100	1.2850	0.070*	
C1	0.4669 (4)	0.4931 (9)	0.6934 (7)	0.114 (3)	
H1B	0.4700	0.4028	0.6543	0.170*	
H1C	0.5149	0.5252	0.7309	0.170*	
H1D	0.4452	0.5503	0.6096	0.170*	
C2	0.4210 (3)	0.4943 (9)	0.8276 (7)	0.0697 (18)	
C3	0.3884 (4)	0.6094 (7)	0.8730 (8)	0.081 (2)	
H3A	0.3945	0.6885	0.8178	0.098*	
C4	0.3475 (3)	0.6136 (6)	0.9950 (7)	0.0662 (17)	
H4A	0.3264	0.6938	1.0224	0.079*	
C5	0.3382 (3)	0.4966 (6)	1.0771 (6)	0.0503 (13)	
C6	0.3695 (3)	0.3769 (6)	1.0334 (7)	0.0634 (17)	
H6A	0.3630	0.2972	1.0871	0.076*	
C7	0.4102 (3)	0.3791 (7)	0.9098 (8)	0.0754 (19)	
H7A	0.4312	0.2994	0.8807	0.090*	
C8	0.1733 (3)	0.3692 (5)	1.0920 (6)	0.0510 (14)	
C9	0.1384 (3)	0.4848 (5)	1.0265 (7)	0.0630 (16)	
H9A	0.1556	0.5690	1.0613	0.076*	
C10	0.0799 (3)	0.4767 (5)	0.9126 (7)	0.0547 (15)	
H10A	0.0573	0.5548	0.8710	0.066*	
C11	0.0539 (3)	0.3519 (6)	0.8589 (6)	0.0531 (14)	
C12	0.0869 (3)	0.2351 (6)	0.9199 (6)	0.0543 (14)	
H12A	0.0690	0.1518	0.8832	0.065*	
C13	0.1475 (3)	0.2419 (5)	1.0376 (6)	0.0449 (12)	
C14	0.1818 (3)	0.1175 (5)	1.1044 (7)	0.0533 (14)	
C15	0.1805 (4)	−0.1223 (5)	1.0982 (8)	0.088 (2)	
H15A	0.1529	−0.1942	1.0445	0.132*	
H15B	0.1786	−0.1294	1.2100	0.132*	
H15C	0.2301	−0.1283	1.0778	0.132*	

### Atomic displacement parameters (Å^2^)

**Table:**

	*U*^11^	*U*^22^	*U*^33^	*U*^12^	*U*^13^	*U*^23^
S	0.0692 (9)	0.0539 (9)	0.0446 (8)	−0.0107 (9)	0.0018 (7)	−0.0062 (8)
Cl	0.0678 (10)	0.0855 (12)	0.0761 (11)	0.0157 (9)	−0.0087 (8)	−0.0023 (9)
O1	0.101 (3)	0.097 (3)	0.041 (2)	−0.019 (3)	−0.020 (2)	0.006 (2)
O2	0.083 (3)	0.052 (3)	0.084 (3)	−0.002 (2)	0.021 (2)	−0.017 (2)
O4	0.087 (3)	0.059 (3)	0.076 (3)	0.005 (2)	−0.023 (3)	0.005 (2)
O5	0.075 (3)	0.036 (2)	0.077 (3)	0.004 (2)	−0.006 (2)	0.004 (2)
N	0.069 (3)	0.057 (3)	0.049 (3)	−0.013 (3)	0.005 (2)	0.009 (2)
C1	0.091 (5)	0.192 (9)	0.058 (4)	−0.041 (6)	0.012 (4)	−0.015 (5)
C2	0.053 (4)	0.107 (6)	0.045 (3)	−0.020 (4)	−0.010 (3)	0.001 (4)
C3	0.089 (5)	0.080 (5)	0.074 (5)	−0.023 (4)	0.005 (4)	0.029 (4)
C4	0.075 (5)	0.049 (4)	0.073 (4)	−0.002 (3)	0.002 (4)	0.013 (3)
C5	0.053 (3)	0.052 (3)	0.043 (3)	−0.006 (3)	−0.006 (2)	−0.005 (3)
C6	0.080 (4)	0.056 (4)	0.053 (4)	−0.003 (3)	0.006 (3)	−0.001 (3)
C7	0.070 (4)	0.089 (5)	0.064 (4)	0.008 (4)	−0.001 (4)	−0.019 (4)
C8	0.055 (4)	0.051 (3)	0.047 (3)	−0.002 (3)	0.007 (3)	−0.008 (3)
C9	0.079 (4)	0.035 (3)	0.075 (4)	0.010 (3)	0.010 (4)	0.008 (3)
C10	0.058 (4)	0.037 (3)	0.068 (4)	0.008 (3)	0.003 (3)	0.007 (3)
C11	0.052 (3)	0.058 (4)	0.049 (3)	0.007 (3)	0.002 (3)	0.008 (3)
C12	0.052 (3)	0.055 (4)	0.058 (3)	−0.005 (3)	0.012 (3)	0.002 (3)
C13	0.054 (3)	0.037 (3)	0.044 (3)	0.001 (3)	0.008 (3)	0.004 (2)
C14	0.068 (4)	0.040 (3)	0.053 (4)	−0.001 (3)	0.011 (3)	0.003 (3)
C15	0.132 (6)	0.032 (4)	0.100 (5)	0.023 (4)	0.019 (5)	0.020 (3)

### Geometric parameters (Å, °)

**Table:**

S—O2	1.434 (4)	C4—H4A	0.9300
S—O1	1.436 (4)	C5—C6	1.395 (7)
S—N	1.634 (4)	C6—C7	1.372 (8)
S—C5	1.740 (5)	C6—H6A	0.9300
Cl—C11	1.736 (5)	C7—H7A	0.9300
O4—C14	1.215 (6)	C8—C9	1.398 (7)
O5—C14	1.331 (6)	C8—C13	1.409 (7)
O5—C15	1.459 (6)	C9—C10	1.363 (7)
N—C8	1.442 (6)	C9—H9A	0.9300
N—H1	0.8600	C10—C11	1.386 (7)
C1—C2	1.511 (8)	C10—H10A	0.9300
C1—H1B	0.9600	C11—C12	1.382 (6)
C1—H1C	0.9600	C12—C13	1.410 (6)
C1—H1D	0.9600	C12—H12A	0.9300
C2—C7	1.370 (8)	C13—C14	1.470 (7)
C2—C3	1.372 (8)	C15—H15A	0.9600
C3—C4	1.363 (9)	C15—H15B	0.9600
C3—H3A	0.9300	C15—H15C	0.9600
C4—C5	1.379 (7)		
			
O2—S—O1	120.2 (3)	C2—C7—C6	122.4 (6)
O2—S—N	109.7 (2)	C2—C7—H7A	118.8
O1—S—N	102.9 (2)	C6—C7—H7A	118.8
O2—S—C5	107.7 (3)	C9—C8—C13	119.1 (5)
O1—S—C5	109.1 (3)	C9—C8—N	122.3 (5)
N—S—C5	106.5 (2)	C13—C8—N	118.5 (5)
C14—O5—C15	117.3 (4)	C10—C9—C8	121.4 (5)
C8—N—S	124.8 (4)	C10—C9—H9A	119.3
C8—N—H1	117.6	C8—C9—H9A	119.3
S—N—H1	117.6	C9—C10—C11	120.0 (5)
C2—C1—H1B	109.5	C9—C10—H10A	120.0
C2—C1—H1C	109.5	C11—C10—H10A	120.0
H1B—C1—H1C	109.5	C12—C11—C10	120.6 (5)
C2—C1—H1D	109.5	C12—C11—Cl	119.9 (4)
H1B—C1—H1D	109.5	C10—C11—Cl	119.5 (4)
H1C—C1—H1D	109.5	C11—C12—C13	120.1 (5)
C7—C2—C3	117.0 (6)	C11—C12—H12A	120.0
C7—C2—C1	120.9 (8)	C13—C12—H12A	120.0
C3—C2—C1	122.1 (7)	C12—C13—C8	118.9 (5)
C4—C3—C2	123.2 (6)	C12—C13—C14	120.0 (5)
C4—C3—H3A	118.4	C8—C13—C14	121.1 (5)
C2—C3—H3A	118.4	O4—C14—O5	121.8 (5)
C3—C4—C5	118.6 (6)	O4—C14—C13	125.2 (5)
C3—C4—H4A	120.7	O5—C14—C13	113.0 (5)
C5—C4—H4A	120.7	O5—C15—H15A	109.5
C4—C5—C6	120.0 (5)	O5—C15—H15B	109.5
C4—C5—S	121.2 (5)	H15A—C15—H15B	109.5
C6—C5—S	118.7 (4)	O5—C15—H15C	109.5
C7—C6—C5	118.7 (6)	H15A—C15—H15C	109.5
C7—C6—H6A	120.7	H15B—C15—H15C	109.5
C5—C6—H6A	120.7		
			
O2—S—N—C8	53.5 (5)	S—N—C8—C13	151.2 (4)
O1—S—N—C8	−177.4 (4)	C13—C8—C9—C10	0.2 (8)
C5—S—N—C8	−62.7 (5)	N—C8—C9—C10	−176.8 (5)
C7—C2—C3—C4	0.8 (9)	C8—C9—C10—C11	−0.1 (8)
C1—C2—C3—C4	−179.1 (5)	C9—C10—C11—C12	−0.1 (8)
C2—C3—C4—C5	−0.1 (9)	C9—C10—C11—Cl	178.5 (4)
C3—C4—C5—C6	−0.5 (8)	C10—C11—C12—C13	0.2 (8)
C3—C4—C5—S	175.0 (4)	Cl—C11—C12—C13	−178.4 (4)
O2—S—C5—C4	13.7 (5)	C11—C12—C13—C8	−0.1 (7)
O1—S—C5—C4	−118.4 (4)	C11—C12—C13—C14	179.0 (5)
N—S—C5—C4	131.2 (4)	C9—C8—C13—C12	−0.1 (7)
O2—S—C5—C6	−170.7 (4)	N—C8—C13—C12	177.0 (4)
O1—S—C5—C6	57.2 (5)	C9—C8—C13—C14	−179.2 (5)
N—S—C5—C6	−53.2 (5)	N—C8—C13—C14	−2.1 (7)
C4—C5—C6—C7	0.5 (8)	C15—O5—C14—O4	−0.4 (8)
S—C5—C6—C7	−175.1 (4)	C15—O5—C14—C13	179.4 (5)
C3—C2—C7—C6	−0.8 (8)	C12—C13—C14—O4	−180.0 (5)
C1—C2—C7—C6	179.1 (5)	C8—C13—C14—O4	−0.9 (8)
C5—C6—C7—C2	0.2 (8)	C12—C13—C14—O5	0.2 (7)
S—N—C8—C9	−31.8 (7)	C8—C13—C14—O5	179.3 (5)

### Hydrogen-bond geometry (Å, °)

**Table:**

*D*—H···*A*	*D*—H	H···*A*	*D*···*A*	*D*—H···*A*
N—H1···O4	0.86	2.07	2.615 (6)	120
C9—H9A···O2	0.93	2.35	3.022 (6)	129
